# Innate immune response adaptation in mice subjected to administration of DMBA and physical activity

**DOI:** 10.3892/ol.2013.1774

**Published:** 2013-12-24

**Authors:** DOUGLAS R. ABDALLA, ANDRÉ ADRIANO ROCHA ALEIXO, EDDIE F.C. MURTA, MÁRCIA A. MICHELIN

**Affiliations:** 1 Oncology Research Institute, Federal University of the Triângulo Mineiro, Uberaba, Minas Gerais, CEP 38100-000, Brazil; 2 Discipline of Gynecology and Obstetrics, Federal University of the Triângulo Mineiro, Uberaba, Minas Gerais, CEP 38100-000, Brazil; 3 Discipline of Immunology, Federal University of the Triângulo Mineiro, Uberaba, Minas Gerais, CEP 38100-000, Brazil

**Keywords:** immune response, cancer, tumor, physical activity

## Abstract

Although there is growing interest in studies that promote the benefits of exercise and the correlation between exercise and fighting cancer, previous studies have not been able to elucidate the underlying mechanisms. The aim of the present study was to investigate cytokine synthesis by peritoneal macrophages in the presence of mammary tumors and the effect of physical activity. Female BALB/c virgin mice (age, eight weeks) were obtained for the present study and divided into four groups: A no tumor/non-trained control group; a no tumor/trained group subjected to swim training; a tumor/non-trained group in which the mice received the carcinogenic drug, DMBA and a tumor/trained group in which the mice were subjected to DMBA and swim training protocols. Following the experimental period, immune cells were collected from the peritoneal fluid, placed in culture medium and stimulated with lipopolysaccharide. The presence of the cluster of differentiation-14 marker and expression of the interleukin (IL)-12 cytokine was assessed by flow cytometry and measured via an enzyme-linked immunosorbent assay. The following cytokines were also identified: Interferon-γ, IL-4, IL-10, IL-12, tumor necrosis factor-α and transforming growth factor-β. Physical activity increased the quantity of IL-12 producing macrophages, whereas the presence of a tumor decreased the quantity of macrophages expressing IL-12. Tumor induction, in the absence of swim training, reduced macrophage-profile 1 (M1) cytokine levels while increasing the presence of macrophage-profile 2 cytokines. Physical activity in mice with tumors resulted in reductions in tumor development and promoted immune system polarization towards an antitumor M1 response pattern profile.

## Introduction

Macrophages in the immune response are considered to be a phagocyte system, which may occasionally be dependent on the state of the host immune reaction (the macrophage response may be influenced for the synthesis of mediators by other immune cells and interaction with other cells), as well as maintenance of the antigen. Proinflammatory classical activation of macrophages is characterized by the secretion of cytokines, such as interferon (IFN)-γ and tumor necrosis factor (TNF)-α, from T helper cells (Th1) and natural killer cells. This activation results in a macrophage population that exhibits enhanced microbicidal performance and increases the secretion of proinflammatory cytokines, which enhance adaptive immunity (1–4).

Conversely, alternative activation is achieved by the presence of interleukin (IL)-4 and −13, which polarize the Th2 response in the macrophages and are important in the immune response in acute or chronic disease processes (5,6).

Currently, macrophages are designated as macrophage-profile 1 (M1) and macrophage-profile 2 (M2), analogous to the division of the Th1 and Th2 profiles. However, M2 macrophages are divided into three subtypes (M2a, M2b and M2c). M2a are stimulated by IL-4 or −13, M2b are stimulated by immune complexes on the Toll-like receptor and the production of proinflammatory cytokines (IL-1β, TNF-α and IL-6), and M2c are stimulated by IL-10 or transforming growth factor (TGF) -β when subjected to anti-inflammatory glucocorticoid hormones (7,8).

Solid tumors recruit macrophages within the microenvironment (tumor-associated macrophages; TAMs) that exhibit a complex association with the neoplastic cells of the tumor. These neoplastic cells perform various roles during tumor development, which are occasionally antagonistic. It was hypothesized that TAM cells presented an antitumor effect as, in a satisfactory environment (when there are immune cells that produce cytokines and other mediators with antitumor profiles), TAMs are capable of causing tumor cell death. However, experimental and clinical studies indicate that rather than promote the neutralization of the tumor, TAMs facilitate angiogenesis, extracellular matrix breakdown and remodeling, and promote tumor cell motility (9–12).

With regard to the role of macrophages in promoting an antitumor response under the influence of physical activity, a study comparing healthy animals versus animals with tumors, identified that exercise resulted in a positive effect on the function of macrophages, which was evidenced by reduced levels of pulmonary metastasis (13). Moreover, the exercise training resulted in greater cytolytic activity *in vitro* (14).

However, few studies have investigated exercise, the function of macrophages and the production of their cytokines in the presence of tumors. In the present study, the immunological profile of mice subjected to DMBA, with or without physical activity, was evaluated.

## Materials and methods

### Experimental groups and tumor induction

Female BALB/c virgin mice (age, eight weeks) were obtained from the Institute for Research in Oncology, Federal University of the Triângulo Mineiro (Uberaba, Brazil) and were group-housed. The present study was approved by the Ethics Committee of the Federal University of Triângulo Mineiro (Uberaba, Brazil; registration no. 160).

The mice were divided into the following our groups (n=14 per group): i) No tumor/non-trained; ii) no tumor/trained (swim training five days/week for eight weeks); iii) tumor/non-trained and iv) tumor/trained (following a matching protocol to group ii). In the tumor groups, the tumors were induced by oral administration of DMBA at a concentration of 1 mg/ml by daily gavage, for six weeks.

### Peritoneal macrophage culture

The mice were euthanized with an overdose of anesthetics, ketamine (50 mg/kg) and xylazine (15 mg/kg) and peritoneal lavage was performed to obtain the macrophages. Three lavages were centrifuged at 290 × g for 10 min at 4°C using RPMI-1640 (Sigma-Aldrich, St. Loius, MO, USA). The cells were counted, resuspended in complete RPMI medium and distributed into 24-well plates at a concentration of 1×10^6^ cells/well to obtain the adherent cells in a 1.0-ml volume, which were subsequently stimulated with 10 μg/ml lipopolysaccharide (LPS). Following 24 h of incubation at 37°C in a 5% CO_2_ atmosphere, the supernatant samples were obtained and the cells were stored at −80°C.

### Flow cytometry

Isolated peritoneal macrophages were placed in 1 ml phosphate-buffered saline (PBS) supplemented with 2 μl protein transport inhibitor (BD GolgiStop^™^; BD Biosciences, Franklin Lakes, NJ, USA) per 3 ml peritoneal macrophage solution, and incubated for ≥20 min at 4°C. The cells were washed with PBS by centrifugation, using the method described above, to remove excess proteins.

Following centrifugation, the cells were resuspended, counted and subjected to extracellular immunolabeling with fluorescent anti-cluster of differentiation (CD)-14 antibody (BD Biosciences, San Diego, CA, USA). The cells were incubated with each antibody for 30 min at 4°C in the dark and washed with PBS to remove excess antibodies. Permeabilization and fixation were performed using BD Cytofix/Cytoperm^™^ solution (BD Biosciences) at 4°C for 20 min in the dark.

The cells were subjected to intracellular immunolabeling using antibodies against IL-12 and TNF-α. Following intracellular labeling, the cells were incubated at 4°C for 30 min in the dark and washed in buffer solution (BD Perm/Wash^™^ Buffer; BD Biosciences) to remove excess labeling molecules. Cell aliquots were resuspended in 500 μl PBS for flow cytometry analysis in a FACSCalibur^™^ cytometer (BD Biosciences).

### Cytokine levels

The presence of cytokines (IFN-γ, IL-4, IL-10, IL-12, TNF-α and TGF-β) in the supernatant samples was measured by an enzyme-linked immunosorbent assay using pairs of monoclonal antibodies (BD OptEIA^™^; BD Biosciences, San Diego, CA, USA). The procedure was performed according to the manufacturer’s instructions.

### Statistical analysis

Data are presented as the mean ± standard error of the mean and the results were analyzed using the analysis of variance test. Proportions were compared using the χ^2^ test and statistical analysis and graphing were performed with GraphPad Prism version 5.0 (GraphPad Software, Inc., La Jolla, CA, USA). P≤0.05 was considered to indicate a statistically significant difference.

## Results

### Profile of immunocompetent cells and expression of cytokines

**Figure 1:**
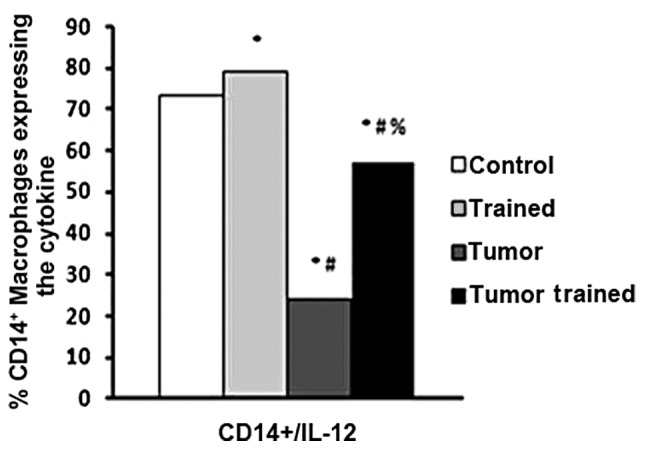
Percentages of CD14^+^/IL-12^+^ double labeled cells per group; ^*^P<0.0001 vs. the no tumor/untrained control group, ^#^P<0.0001 vs. no tumor/trained group and ^%^P<0.0001 vs. the tumor/non-trained group. Comparisons were performed using the χ^2^ test. CD, cluster of differentiation; IL, interleukin; control, no tumor/non-trained; trained, tumor/trained; tumor, tumor/non-trained; tumor trained, tumor/trained.

*Ex vivo* CD14/IL-12 double-labeling experiments identified that physical activity increased the quantity of CD14^+^/IL-12^+^ cells, compared with that of the no tumor/non-trained control group (P<0.0001; Fig. 1). Furthermore, mice in the tumor/non-trained group exhibited a sharp reduction in CD14^+^/IL-12^+^ cell frequency (P<0.0001), when compared with the control group. However, subjecting the mice with tumors to physical training attenuated the tumor-induced reduction, as tumor/trained mice exhibited significantly greater quantities of double-labeled cells than mice in the tumor/non-trained group (P<0.0001; Fig. 1). There were no group differences observed in CD14^+^/TNF-α^+^ double-labeled cells (data not shown).

### Production of cytokines in the supernatant of peritoneal macrophage cultures

**Figure 2:**
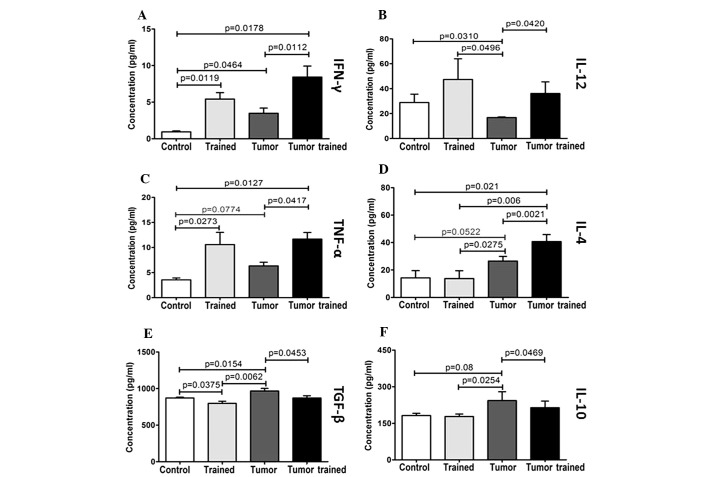
Concentrations of (A) IFN-γ, (B) IL-12, (C) TNF-α, (D) IL-4, (E) TGF-β and (F) IL-10 per group, obtained from 24-h cultures with peritoneal macrophages. IFN, interferon; IL, interleukin; TNF, tumor necrosis factor; TGF, transforming growth factor; control, no tumor/non trained; trained, non tumor/trained; tumor, tumor/non trained; tumor trained, tumor/trained.

Cultures of macrophages, from the mice in the groups that were trained, exhibited greater concentrations of IFN-γ than the cultures obtained from the sedentary groups (P=0.0119, no tumor/trained vs. no tumor/non-trained and P=0.0112, tumor/trained vs. tumor/non-trained; Fig. 2A). The presence of the tumor alone was sufficient to increase the synthesis of IFN-γ (P=0.0464, tumor/non-trained vs. no tumor/non-trained). The combination of the tumor and training markedly induced the expression of IFN-γ relative to the no tumor/non-trained control group (P=0.0178).

As with Th1 cytokines, after 24 h of culturing, there was an increase in levels of IL-12 in the trained groups and a decrease in the group with a tumor alone (Fig. 2B). Relative to the no tumor/non-trained control group, no tumor/trained mice yielded increased levels of IL-12 (P=0.0310) and tumor/non-trained mice yielded reduced levels of IL-12 (P=0.0496). Conversely, physical activity opposed this effect, as tumor/trained mice exhibited greater levels of IL-12 than tumor/non-trained mice (P=0.0420).

TNF-α synthesis followed IL-12 production (Fig. 2C); either physical activity or the presence of a tumor increased TNF-α expression compared with the no tumor/non-trained control group (P=0.0273 and P=0.0127, respectively). Exercise training in mice with tumors further increased TNF-α expression beyond that which was observed in the tumor/non-trained group (P=0.0417).

Training alone did not significantly affect IL-4 levels (Fig. 2D); by contrast, the presence of a tumor tended to increase the IL-4 concentration. IL-4 levels in the tumor/non-trained group increased relative to the control group (P=0.0552) and were significantly greater than the levels observed for the no tumor/trained group (P=0.0275). Practicing physical activity in combination with the presence of a tumor produced IL-4 levels that were significantly higher than the IL-4 levels observed in each of the three other groups (P=0.021 vs. no tumor/non-trained; P=0.006 vs. no tumor/trained; P=0.0021 vs. tumor/non-trained).

Conforming to the immunosuppressive cytokines, relative to the no tumor/non-trained control group, TGF-β concentration (Fig. 2E) was increased after 24 h of culture in the isolated presence of a tumor (P=0.0154); however, it was decreased in the no tumor/trained group (P=0.0375). TGF-β concentration was also lower in the no tumor/trained group than in the tumor/non-trained group (P=0.0062). Furthermore, training resulted in a reduction of TGF-β concentration in cultures from mice with tumors (P=0.0453, tumor/trained vs. tumor/non-trained).

Over a 24 h time period, the IL-10 levels (Fig. 2F) in the no tumor/trained group were analogous to IL-10 levels in the no tumor/non-trained control group and significantly lower than IL-10 levels in the tumor/non-trained group (P=0.0254). In the presence of the tumor, implementing physical activity attenuated the expression of IL-10 (P=0.0469 tumor/trained vs. tumor/non-trained) towards control group levels (P>0.05, tumor/trained vs. no tumor/non-trained; Fig. 2F).

**Figure 3:**
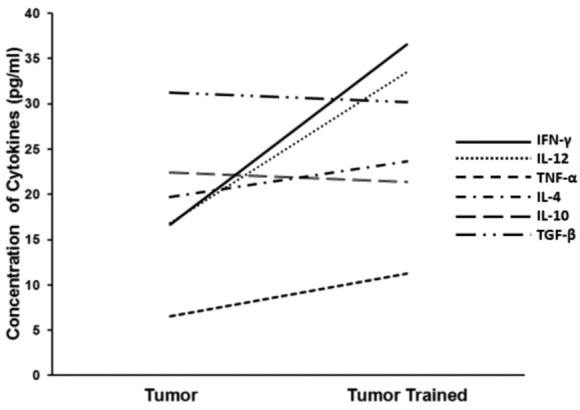
Linear trends for the concentration of samples that expressed Th1 cytokines versus Th2 cytokines or regulatory T cell profile cytokines for the tumor/non-trained group vs. the tumor/trained group. IFN, interferon; IL, interleukin; TNF, tumor necrosis factor; TGF, transforming growth factor.

To observe the changes resulting from performing physical activity, a tendency analysis to compare the M1 cytokines (IFN-γ, TNF-α and IL-12) to the M2 cytokines (IL-4, IL-10 and TGF-β) was conducted. The tumor/trained group exhibited greater concentrations of the three M1 cytokines than the tumor/non-trained group, whereas the trends of the M2 cytokines were less pronounced (Fig. 3). Two M2 cytokines (IL-10 and TGF-β) were marginally greater in the tumor/non-trained group than in the tumor/trained group and one cytokine (IL-4) was marginally greater in the tumor/trained group than in the tumor/non-trained group.

## Discussion

The tumors may affect how the functionality of macrophages change via modification of the microenvironment and thus the immune response, in such a way that the immune system itself is used as a tumor escape mechanism. Therefore, understanding how tumor cells interfere with the action of macrophages and investigating the potential application of this information, may be via identification of the soluble or inhibitory factors that permit the tumors to change the plasticity of the macrophages (15).

To enable the macrophages to act efficiently, IL-12 expression is important and, in the presence of tumors, its expression is correlated with increased survival rates (16). Thus, in the present study it was identified that, in the *ex vivo* system and with the production of IL-12 in the supernatant of the macrophage culture, physical activity was capable of optimizing the expression of IL-12 in the macrophages. Trained mice exhibited greater percentages of CD14^+^ cells expressing IL-12 than the non-trained groups, particularly with the non-trained group that underwent chemical carcinogenesis with DMBA. The results of the present study, therefore, indicate that physical activity is a significant factor that is capable of modulating the immune system and aiding in the antitumor response. The activity of the Th1-pattern cytokines analyzed in the present study, such as IFN-γ and TNF-α, reinforced this hypothesis.

Kizaki *et al* (17) analyzed two groups of mice, which were sedentary or moderately trained (50–75% VO_2_max; 30 min of stair climbing at 18 m/min, 5 days/week for three weeks). It was observed that the trained group exhibited increased concentrations of IFN-γ, TNF-α and nitric oxide, and a reduction of IL-10. Furthermore, the synthesis of cytokines was not observed to be correlated with the quantity of adrenergic β receptors. Thus, Kizaki *et al* (17) demonstrated that the adaptation of macrophages to moderate exercise improved microbicidal activity and the capacity for a Th1-type response. These data corroborate the observations of the present study, in which mice in the no tumor/trained and tumor/trained groups exhibited higher levels of IFN-γ and TNF-α, as well as IL-12, and diminished levels of IL-10 and TGF-β. Thus, by characterizing these macrophages as M1, it may be inferred that these cells were capable of promoting a positive antitumor response.

Lu *et al* (18) verified that chronic physical activity improved the antitumor activity of macrophages. When macrophages were placed in a culture with IFN-γ and LPS, greater quantities of cytolytic macrophages were observed in the trained groups, independent of the age of the animals. Woods *et al* (19) confirmed this outcome.

Bombarda *et al* (20) investigated the effect of a session of exercise, performed below the anaerobic threshold, on the function of neutrophils and circulating monocytes in Wistar rats. The functional activity of circulating phagocytes was evaluated using a *Saccharomyces cerevisiae* phagocytosis assay and a nitro blue tetrazolium (NBT) test. No statistically significant difference was identified between the groups with regard to the total and differential number of leukocytes. However, the neutrophils in the groups that underwent training phagocytosed an increased number of *S. cerevisiae* and exhibited greater efficiency in reducing NBT than the control group. Therefore, exercise performed at an intensity below the anaerobic threshold was sufficient to increase the phagocytic and microbicidal activity of the neutrophil in an animal model.

Such findings indicate that macrophages may mediate immune system defense against tumors. Early liberation of IFN-γ contributes to the differentiation of T cells from Th1 cells. Thus, the initial production of IFN-γ, IL-12 and TNF-α is significant in generating innate immunity and M1 macrophages, as well as improving adaptive defenses against infections. Conversely, IL-4 (an M2 cytokine) and the production of TGF-β promotes a change from Th1 to Th2 or Tregs, suppressing antitumor resistance, thus indicating that low production of IL-4, IL-10 and TGF-β, as seen in the peritoneal macrophages of trained mice, may contribute to the Th1-type immune response against tumors (21,22).

As identified in previous studies, where the profile of T helper lymphocytes in animals with tumors subjected to physical activity was evaluated and a greater expression of Th1-pattern cytokines and a reduction in the expression of Th2 cytokines was demonstrated (23), the data from the present study identified that trained mice and mice with a tumor that practice physical activity exhibit an M1 profile. By contrast, mice with a tumor that remain sedentary have an M2 profile. Thus, regular physical activity is advised for patients with cancer, alongside conventional therapies (such as chemotherapy, radiation treatment and surgery) and in conjunction with novel therapies, such as immunotherapies. However, further investigation is required to standardize training regimes and the frequency, intensity and methods of delivering them in order to verify the point at which physical activity becomes beneficial, or to establish whether too much activity may damage the immune system and favor the development and aggravation of tumors.

In conclusion, inducing tumors in sedentary mice reduced the cytokine synthesis of M1 macrophages and increased the presence of M2 macrophages. However, practicing physical activity in the presence of a tumor promoted a reduction in tumor development and polarized the immunological response in the direction of the antitumor M1 profile.
